# Dataset on the Impact of GO-NGO Support on Crop Intensification and Food Security in Bangladesh

**DOI:** 10.1016/j.dib.2018.03.020

**Published:** 2018-03-08

**Authors:** Md. Monirul Islam, Arifa Jannat, Aurup Ratan Dhar

**Affiliations:** a Department of Agricultural Economics, Bangladesh Agricultural University, Mymensingh-2202, Bangladesh; b Institute of Agribusiness & Development Studies, Bangladesh Agricultural University, Mymensingh-2202, Bangladesh

**Keywords:** Crop intensification, GO-NGO support, Food security, Bangladesh

## Abstract

The data used in this article elucidated crop intensification and farmers’ food security status through GO-NGO support in Bangladesh. A total of 200 farmers (100 from non-supported and 100 from GO-NGO supported) were selected for data collection using purposive sampling technique. The collected data showed that GO-NGO support has a significant impact on changes in agricultural enterprises. Majority (63.3%) of the households belong to the low intensity category for non-supported farmers. In case of GO-NGO supported farmers, majority (73.3%) of the households belong to the high intensity category. The food security indices values showed that the food security index for non-supported farm households was 0.97 and for GO-NGO supported farm households, it was 1.07.

**Specifications Table**

**Table:** 

Subject area	*Agriculture, Economics*
More specific subject area	*Crop intensification and food security*
Type of data	*Table, text file, figure*
How data was acquired	*Field survey*
Data format	*Analyzed*
Experimental factors	*Not applicable*
Experimental features	*Not applicable*
Data source location	*Belkuchi and Chauhali upazila of Sirajganj district, Bangladesh*
Data accessibility	*The data are available with this article*
Related research article	*Not applicable*

**Value of the Data**

• *A number of studies have been conducted on economic, environmental and livelihood prospect of char areas in Bangladesh but there is no specific study on existing farming practices, crop intensification and food security aspects in these areas.*
• *This study will provide valuable information that may be functional both of different levels of GOs and NGOs in order to formulate appropriate policy and intervention strategy for the improvement of the char people in Bangladesh.*
• *This data allows other researchers to extend the statistical analyses.*

## 1 Data

**Fig. 1:**
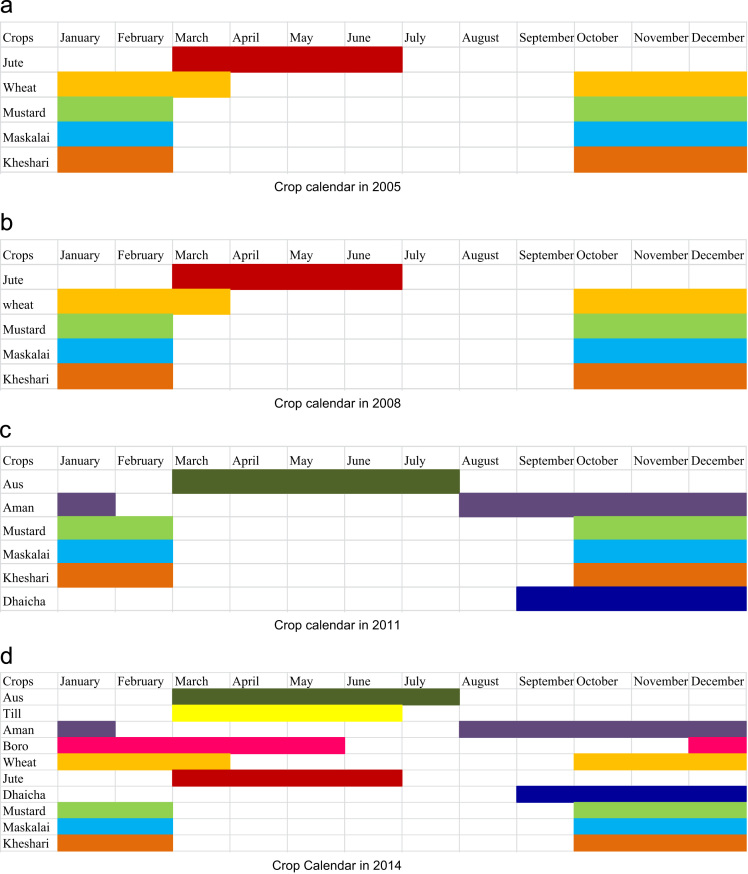
Crop calendar over the years in the study areas. (a) Crop calendar in 2005. (b) Crop calendar in 2008. (c) Crop calendar in 2011. (d) Crop Calendar in 2014.

**Fig. 2:**
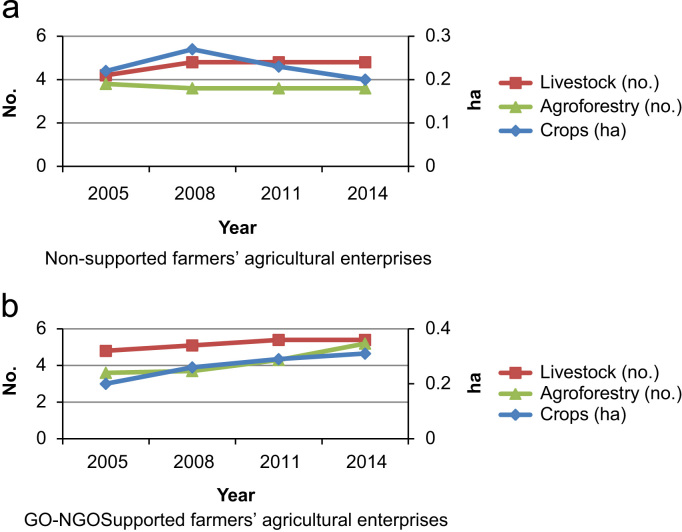
Changing agricultural enterprises of the farmers. (a) Non-supported farmers’ agricultural enterprises. (b)GO-NGO Supported farmers’ agricultural enterprises.

**Fig. 3:**
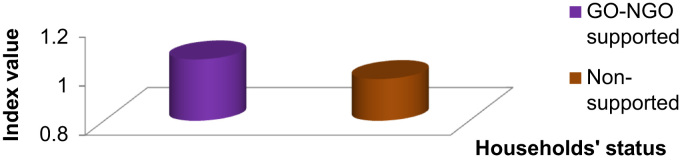
Food security status of the households.

The dataset of this article provides information on the potential impact of GO-NGO support services on crop intensification and food security status of some selected char areas in Bangladesh. The Fig. 1, Fig. 2, Fig. 3 show the changing scenario of agricultural enterprises through crop calendar due to support provided by the different national GOs and NGOs. Table 1, Table 2 show actual differences of crop intensification and changing agricultural practices in the research areas.

**Table 1:** Changes in agricultural enterprises.

Outcome variables	Non-supported farmers	GO-NGO supported farmers	Difference
Crops			
Crops in 2005	0.22	0.20	-0.20
Crops in 2008	0.27	0.26	-0.01
Crops in 2011	0.23	0.29	0.06
Crops in 2014	0.20	0.31	0.11
Difference in 2008-2005	0.05	0.06	0.01
Difference in 2011-2005	0.01	0.09	0.08
Difference in 2014-2005	-0.02	0.11	0.13
Livestock			
Livestock in 2005	4.23	4.80	0.57
Livestock in 2008	4.80	5.13	0.33
Livestock in 2011	4.80	5.43	0.63
Livestock in 2014	4.80	5.35	0.55
Difference in 2008-2005	0.57	0.33	-0.24
Difference in 2011-2005	0.57	0.63	0.06
Difference in 2014-2005	0.57	0.55	-0.02
Agroforestry			
Agroforestry in 2005	3.80	3.63	-0.17
Agroforestry in 2008	3.63	3.70	0.07
Agroforestry in 2011	3.63	4.33	0.70
Agroforestry in 2014	3.63	5.15	1.52
Difference in 2008-2005	-0.17	0.07	0.24
Difference in 2011-2005	-0.17	0.70	0.97
Difference in 2014-2005	-0.17	1.52	1.69

**Table 2:** Levels of crop intensification of farming households.

Households category		No. of respondents	Min	Max	Mean
Non-supported farmers	High intensity	11(37.7%)	14.5	38.5	27.5
	Low intensity	19 (63.3%)	4.5	32.0	19.6
	All households	30 (100.0%)	6.5	38.5	23.1
GO-NGO supported farmers	High intensity	22 (73.3%)	24.5	48.5	37.5
	Low intensity	08 (27.7%)	4.5	23.0	15.4
	All households	30 (100.0%)	11.5	43.5	29.1

Note: Figures within the parenthesis indicate percentage of total respondents.

## 2 Experimental Design, Materials and Methods

In Bangladesh, most of the *char* dwellers are involved in various kinds of agricultural activities and their farming systems are different from the mainland. Crop intensification is defined as the increased average inputs of labour or capital on a smallholding, either cultivated land alone, or on cultivated and grazing land for the purpose of increasing the value of output per hectare [1].

On an average, 5 percent (10 million) person lives on the *char* areas and mainly depends on agricultural activities [2]. A number of social protection interventions such as social safety net programmes, various training facilities and soft loan schemes during the lean period, awareness campaign, introduction of new cropping patterns, various saving programmes, introduction of different income generating activities and assistance upon them, etc. have been providing by the government and non-government organizations to the poorest households in *char* areas. Cropping pattern of *char* areas have been changing year to year due to natural calamities such as river erosion, flood, drought, etc. Sometimes excess flood affects the farming systems in *char* areas. As a result, the cropping pattern of *char* areas varies widely. The changing cropping patterns in *char* areas are shown in Fig. 1(a, b, c and d) with the help of crop calendars. The entire crop calendars picturize that cropping pattern in *char* areas have been changing from the years 2005 to 2014. In 2005, the average number of crops produced in *char* areas was 5. It was same in the year of 2008. In 2011 and 2014, it was 6 and 10, respectively.

Fig. 2(a) shows that amount of land under cultivation decreased for non-supported farmers; and it was 0.22 ha and 0.20 ha in 2005 and 2014, respectively. On the contrary, GO-NGO supported farmers has a positive trend for land cultivation (from 0.20 ha in 2005 to 0.31 ha in 2014) which is found from Fig. 2(b). Similar trend was also found in case of both number of livestock and agroforestry enterprises for the GO-NGO supported farmers than the non-supported one. From Fig. 2 it is clear that GO-NGO support has a significant impact on changes in agricultural enterprises. Reference [3] analyses the development of economic measures on a sample of agricultural enterprises in the Czech Republic in 2006–2010. Compared to 2009, the situation of agricultural enterprises improved in 2010. Primarily, total production after its considerable decline in 2009 increased only slightly and reached a level of 2006 in the average enterprise.

In most of the cases, there is a positive and statistically significant difference over the years from 2005 to 2014 for GO-NGO supported farmers while for non-supported farmers, the results vary from year to year as well as enterprise to enterprise. The results suggested that the GO-NGO support and services had both short (2008 to 2005) and long-term (2014 to 2005) impacts on increasing agricultural enterprises of the GO-NGO supported farmers in *char* areas, that is, the GO-NGO support and services made it possible for the *char* households to enrich their agricultural resources (Table 1).

The high intensity of farming households had the maximum and mean crop intensity scores of 38.5 and 27.5 for non-supported farmers and for GO-NGO supported farmers those were, 48.5 and 37.5, respectively which were higher than those of the low intensity households in *char* areas (Table 2).

Majority (63.3%) of the households belong to the low intensity category while the remaining 37.7% are high intensity households for non-supported farmers. In case of GO-NGO supported farmers, majority (73.3%) of the households belong to the high intensity category while the remaining 27.7% are low intensity households. Reference [4] estimated cropping intensification, the levels of technical efficiency of 252 maize-based farming households in Southern-Guinea Savannah (SGS) of Nigeria and showed that the crop production intensity scores among the farming households ranged between 5.5 and 38.50 with a mean score of 23.13. The high intensity farming households had the maximum and mean crop intensity scores of 38.50 and 27.47 respectively, which were higher than those of the low intensity households. Reference [5] revealed that crop intensification program (CIP) in Rwanda has successfully convinced farmers by explaining the various advantages of land consolidation among them that increased land and crop productivity amazingly. As a result, the consolidated use of land area under these crops has been increased from 28788 ha in 2007 to 254000 ha in 2010 and 502916 ha in 2011.

The food security index and other related food security measures such as, food insecurity gap/surplus index and head count ratio have been constructed separately for both non-supported and GO-NGO supported farmers. The food security index for food secured households was 1.13 for non-supported farmers and for GO-NGO supported farmers, it was 1.33 whereas for food insecured households, it was 0.84 and 0.80, respectively. Based on the recommended daily calorie intake of 2,122 kcal, it is observed that 57.0 percent of households were food secured in case of non-supported farmers and for GO-NGO supported farmers it was 60.0 percent (Fig. 3).

Average calorie intake of food secured households were 2,390.55 kcal and 2,818.11 kcal for non-supported and GO-NGO supported farmers, respectively which are higher than the national average calorie intake (i.e., 2,122 kcal). The food security gap or surplus index shows that the food secure households exceeded the food poverty line by 13 percent, while food insecure households fell short of the required calorie intake by 16 percent for non-supported farmers and for GO-NGO supported it was 33.0 and 20.0 percent, respectively [6].

Reference [6] carried out a study on adapting an experiential scale to measure food insecurity in urban slum households of India. A nine-item experience-based food security scale was constructed by adapting the United States Household Food Security Survey Module, according to which 15.4 percent of the households are food insecure. Findings also indicated that multi-sectoral interventions were required to tackle the problem of urban food insecurity. Reference [7] assessed the contribution of improved rice varieties in poverty reduction and food security in Sub-Saharan Africa. A positive impact of improved varieties on food security and poverty reduction was observed over the period 2000-2014. In addition, the rate of adoption of these varieties increased over these years and this increase was more significant after the 2008 food crisis. These trends could be enhanced by addressing production constraints and certified seed bottlenecks. Also, the obtained results are consistent with the results from previous studies by Akhi et al. [8].
